# Management strategies to de-implement low-value care—an applied behavior analysis

**DOI:** 10.1186/s43058-022-00320-3

**Published:** 2022-06-25

**Authors:** Sara Ingvarsson, Henna Hasson, Hanna Augustsson, Per Nilsen, Ulrica von Thiele Schwarz, Ingunn Sandaker

**Affiliations:** 1 Procome Research Group, Medical Management Centre, Department of Learning, Informatics, Management and Ethics, Karolinska Institutet, 171 77 Stockholm, SE Sweden; 2 Unit for Implementation and Evaluation, Center for Epidemiology and Community Medicine (CES), Stockholm Region, 171 29 Stockholm, SE Sweden; 3 Department of Health, Medical and Caring Sciences, Division of Public Health, Linköping University, Linköping, Sweden; 4 School of Health, Care and Social Welfare, Mälardalen University, Box 883, 721 23 Västerås, Sweden; 5 SCBE Research Group, Department of Behavioral Science, Faculty of Health Sciences, Oslo Metropolitan University, St. Olavs plass, P.O. Box 4, NO-0130 Oslo, Norway

**Keywords:** De-implementation, Low-value care, Primary health care, Physicians, Applied behavior analysis, Three-term contingency, Rule-governed behavior, Sweden

## Abstract

**Background:**

There is a lack of knowledge about management strategies being used to de-implement low-value care (LVC). Furthermore, it is not clear from the current literature what mechanisms are involved in such strategies and how they can change physicians’ behaviors. Understanding the mechanisms is important for determining a strategy’s potential impact. Applied behavior analysis focuses on processes involved in increasing and decreasing behaviors. Therefore, the aim of this study is to understand what management strategies are being used to de-implement LVC and the possible mechanisms involved in those strategies, using concepts from applied behavior analysis.

**Method:**

We applied a qualitative study design using an inductive approach to understand what management strategies are in use and then employed applied behavior analysis concepts to deductively analyze the mechanisms involved in them.

**Results:**

We identified eight different management strategies intended to influence LVC. Five of the strategies were developed at a regional level and had the potential to influence physicians’ LVC-related behaviors either by functioning as rules on which LVC to de-implement or by initiating local strategies in each health care center that in turn could influence LVC practices. The local strategies had a stronger potential for influencing de-implementation.

**Conclusion:**

Both strategies at a systemic level (regional) and on a local level (health care centers) must be considered to influence LVC-related behaviors. Strategies at the center level have a specific opportunity to impact LVC-related behaviors because they can be tailored to specific circumstances, even though some of them probably were initiated as an effect of strategies on a regional level. Using applied behavior analysis to understand these circumstances can be helpful for tailoring strategies to reduce LVC use.

**Supplementary Information:**

The online version contains supplementary material available at 10.1186/s43058-022-00320-3.

Contributions to the literature

– Presents knowledge about potential management strategies that health care organizations can use to support professionals in reducing LVC
– Describes the mechanisms involved in various strategies to de-implement LVC, i.e., the processes by which strategies produce desired effects
– Illustrates an example of how central concepts from applied behavior analysis can be used as a method for understanding these mechanisms

## Background

Low-value care (LVC) is a well-known problem within health care where resources are used for practices other than those based on the best evidence [1–4]. The prevalence of LVC varies among studies, which report that anywhere from 14% [5] to 30% of all practices provide LVC [6]. Common examples of LVC are prescribing non-indicated antibiotics, prescribing potentially inappropriate medication for the elderly, and ordering unnecessary imaging and low-value lab tests [7].

Several studies have been conducted to identify the determinants for LVC usage and de-implementation [7], and they describe a wide range of influencing factors. Examples of determinants include those related to the practice itself (e.g., conflicting guidelines, LVC easier to distribute than evidence-based practice), the individual clinician’s knowledge and attitudes, organizational factors, societal views on the practice, and patients’ expectations and demands. Differences have been found in the prevalence of LVC among organizations [8], indicating that management strategies may play an important role in LVC use. Examples of strategies include quality monitoring, process change, and financial incentives. However, although differences in LVC use among organizations are known in the literature, knowledge is scarce about potential management strategies that health care organizations can use to support professionals in reducing LVC [9]. Furthermore, there is a paucity of knowledge about what mechanisms are involved in various strategies that can support LVC de-implementation, i.e., the processes by which strategies produce the desired effect [10].

Strategies to achieve LVC de-implementation need to decrease certain behaviors among professionals, e.g., ordering unnecessary tests and prescribing unnecessary medications. However, LVC de-implementation strategies may also require increasing other behaviors. Examples of behaviors that might need to increase are ordering diagnostic tests to rule out the need for antibiotics or anti-malaria medication [11] and communicating with patients on the reason for not using a given LVC practice [12]. Thus, to fully understand the management strategies for de-implementation, there is a need to understand both the behaviors that need to decrease and those that need to increase.

Few behavior change theories distinguish between increasing and decreasing behaviors [13]. However, the operant learning theory explicitly distinguishes between strategies for increasing and decreasing behaviors [13, 14]. The contemporary application of the operant learning theory is applied behavior analysis, which has been used in health care for a variety of purposes (e.g., increased staff attendance [15], improved compliance with safety routines [16], increased emergency department efficiency [17], improved compliance to routines [18, 19]). However, to the best of our knowledge, applied behavior analysis has yet to be used to understand the management strategies for LVC de-implementation, despite its potential usefulness for understanding how and why strategies influence LVC-relevant behaviors.

### Central theoretical concepts within applied behavior analysis

Applied behavior analysis focuses on behavior, with the three-term contingency and rule-governed behavior as two of its central concepts. The three-term contingency includes analyzing a certain behavior’s consequences and antecedents to explain that behavior. Thus, instead of explaining a behavior based on people’s cognitions (e.g., beliefs, intentions, and motivations), applied behavior analysis emphasizes how behaviors are established, maintained, and extinguished as a function of their relation to their environment [20, 21]. The probability of a behavior occurring depends on the functional relation between the behavior and its immediate consequences [22]. For instance, the three-term contingency can provide an explanation for why patients influence the use of LVC, as suggested in the literature [9]. Patients’ anxiety about their own health or requests for a specific LVC practice can be understood as antecedents for a physician’s behavior to deliver a practice. A favorable reaction from a patient (e.g., relief) can function as a consequence that increases the likelihood that the physician will use the same practice again in the future. Thus, if patient interactions are the contingencies that reinforce physicians’ LVC-using behaviors, strategies to de-implement LVC need to change those contingencies. This can be done by increasing the effort needed to engage in the LVC-related behavior that is associated with the reinforcing consequence (i.e., by increasing response effort). It can also be done by changing the antecedents and consequences for behaviors related to not using LVC, so that these contingencies more strongly influence the physicians’ behaviors (i.e., differential reinforcement) [23, 24].

Rule-governed behavior is a concept within behavior analysis. As described above, *three-term contingency* is a simple description of how antecedents, behaviors, and their consequences are interrelated. However, three-term contingency is insufficient to explain behaviors that have been established without exposure to real-life antecedents and consequences [25]. These behaviors can be shaped by instructions in which the consequences of the behaviors are stated. Rules are suitable for situations where learning by consequences is too time-consuming or dangerous (e.g., based on patient safety). From an early age, we learn that rules—the description of a reliable relation between a behavior and a consequence—are smart to follow. “Do not touch the stove, as you may burn yourself.” “Watch out before crossing the road, or you may be hit by a car.” These are both warnings that describe a relation between a behavior and a potential threat. We also have rules that describe relations between a behavior and a potential gain, such as “Do your homework, and receive good grades” or “Being generous to others will give you benefits in the long run.” Whether as children, or later in life as employees, people follow rules depending on the complex interplay of, and sometimes concurrent relations between, behaviors and the environment.

Once individuals have learned to follow rules, rule-following becomes a generalized behavior, making it easier to follow new rules [26]. This might, however, be a double-edged sword. While it seems smart to follow rules to avoid negative experiences others may already have been exposed to, research indicates that rule-following may make us less sensitive to real contingencies in situations when this would be appropriate. When a rule is established as governing a behavior, it can lead to less flexible behavioral repertoires that yield insensitivity and unresponsiveness to real-life consequences that ought to influence our behavior. This could partially explain why certain LVC practices continue despite the introduction of new guidelines advising against them [26].

In sum, there is a lack of knowledge about management strategies being used to de-implement LVC. Furthermore, it is not clear from the current literature what mechanisms are involved in such strategies and how they can change physicians’ behaviors. Understanding the mechanisms is important for determining a strategy’s potential impact.

Therefore, the aim of our study was to understand what management strategies are being used to de-implement LVC and the possible mechanisms involved in those strategies using applied behavior analysis.

## Methods

We applied a qualitative explanatory study design using an inductive approach to understand what management strategies are used and then employed applied behavior analysis to deductively analyze the mechanisms involved in them.

### Study setting

We aimed for a great variety of participating organizations to obtain rich information about different management strategies. We conducted our study within public and private primary care practices in Stockholm, Uppsala, and Halland counties in Sweden. Swedish primary care is part of the tax-funded health system, which is governed and managed by 21 counties across Sweden. The private and public centers are funded and governed according to the same rules but can differ in organization and management practices.

### Recruitment and participants

We used a combination of purposeful and snowball sampling approaches to recruit senior managers, line managers, and other key stakeholders. In the first step, we aimed to purposefully include senior managers and other key stakeholders at an overall level (e.g., people who were responsible for continuous staff education or quality assurance) in each organization. In the second step, we aimed to include line managers and stakeholders in the same organization at a more operative level (e.g., physicians responsible for medical processes). This process relied on snowball sampling via the key stakeholders contacted in the first step. We contacted senior managers and stakeholders at an overall level via e-mail. A total of four key stakeholders were contacted, all of which agreed to participate. In the second step, the senior managers recommended and contacted operative level managers and stakeholders in their own organizations. A total of six managers and two operative stakeholders accepted to participate. All respondents who accepted the invitation and consented to study participation were included.

In sum, a total of 12 managers were included (1 senior manager, 3 overall key stakeholders, 6 line managers, and 2 key stakeholders on an operational level). Three respondents were from private providers and nine from public providers. Since the participants held characteristics that were specific for the study aim, 12 participants were estimated to conclude enough information power for the study [27]. The proportion of private versus public centers was somewhat lower than the average proportion in Sweden (approximately 44% [28]), but no comparison was made between private and public centers. The number of private centers was considered to be sufficient.

### Data collection

Data was collected through semi-structured individual interviews. We first interviewed overall-level respondents to get information on which strategies were being used to de-implement LVC in their councils. Second, we interviewed respondents who worked at a more operative level to capture their experience of the different strategies that they perceived as influencing LVC use. The interviews focused on strategies influencing the general use of LVC and strategies influencing physicians’ use of LVC. We chose to talk about physicians because they are the professional group responsible for prescribing most practices within Swedish primary health care, e.g., lab tests, examinations, and medications. All interviews were performed via Zoom and recorded. The recordings were transcribed verbatim.

### Data analysis

We analyzed the data in two steps. First, we used an inductive process to analyze which management strategies were used and how well they were perceived to work. Then, we used applied behavior analysis to analyze the mechanisms involved in the described strategies.

The inductive analysis was carried out as a qualitative content analysis [29]. The first author identified meaning units and coded them through a process of line-by-line coding in nVivo. The codes were then grouped into preliminary categories and subcategories. Parallel to this process, memos were written to capture preliminary ideas and thoughts around the meaning of the data. In the next step, the first author further expounded on these categories and memos together with the last author. Then, the two authors tested these ideas by returning to the material and validating or discarding the results. Throughout the analysis process, both authors discussed and validated the findings.

In the “9” section, we illustrate the categories through rich quotes from the interviews using “()” when text has been added. We made minor modifications to some quotations to make them clearer and easier to understand without altering the meaning of the statements.

We then analyzed the strategies found in the inductive analysis to identify functions using the three-term contingency and rule-governed behavior concepts from applied behavior analysis. Each strategy was related to these concepts; we interpreted how likely it was that they would either influence the three-term contingencies for the physicians’ LVC-related behaviors or function as rules governing the physicians’ behaviors. We used answers related to how well the interviewees perceived the different strategies to function and examples they gave of how these strategies had influenced their centers’ work in our deductive analysis. The data analysis was conducted and reported in accordance with the consolidated criteria for reporting qualitative research (COREQ). Adherence to the COREQ checklist is available in Additional file 1: Table S1.

## Results

### Inductive analysis

The inductive analysis revealed eight types of management strategies that were perceived as influencing LVC use. Five of these were management strategies at the regional level: scorecards, clinical decision support, lectures, quality assurance systems, and financial systems. Three strategies: process strategies, locally held lectures, and discussions about guidelines, were decided at the center level.

#### Scorecards

Most centers used scorecards to track their own performance. Some respondents believed this had influenced LVC, whereas others did not think so. Some key performance measures were decided regionally, either by those financing health care or by the regional management teams, while others were added by the centers themselves. Most key measures had a general goal of improving care—e.g., patient safety—but some were described as more specifically related to LVC, e.g., number of antibiotic prescriptions and cost of lab tests per patient visit.

We use it to follow up guidelines, routines, and other templates. So, I believe you can say that it has an influence. Sure, we haven’t had a specific focus on it (reducing LVC) or set a clear agenda for it. But at least indirectly. (IP4)

#### Guidelines

Guidelines were also a possible way to influence LVC use, with differences between centers concerning which guideline was used. The guidelines were described in terms of written instructions on how to manage the different types of diagnoses within the health care system. Region Stockholm administrators developed their own guidelines based on a combination of local knowledge and national guidelines. The regional guidelines were published on a website (viss.nu) and included both guidelines on how to manage different types of diseases and how the responsibility should be divided between primary care and specialist care. Participants from the other two counties did not describe similar regional guidelines but used either a national decision support system called Internet Medicine or used the guidelines developed by Region Stockholm. Like scorecards, the guidelines had a general goal of improving care, even though some of the guidelines were possible to relate to LVC de-implementation.

The centers differed in their perceptions of whether the guidelines influenced LVC use. Guidelines were described as being written by specialists and designed for specific diseases which led to more LVC. Patients’ symptoms could be due to several diseases, meaning that if they were to follow all the guidelines, it would lead to unnecessary lab tests, examinations, and medications:

But in many of the groups, there is no primary care physician and only other specialists; and that leads to more test ordering and examinations, since they are used to another patient population. (IP3)

At the same time, some guidelines were perceived to contribute to less LVC, as they recommended a stepwise approach to lab tests and examinations and encouraged the physicians to limit their use of certain treatments.

You do not order the full set of tests at once, and that by definition means that you will order fewer lab tests – that you will order the right lab tests, and that you will find the right track for your assessment (of the patient) sooner. (IP1)

Still, others perceived that the guidelines had no impact on their use of LVC because none of the guidelines was written specifically to target LVC:

It doesn’t matter how many decision supports systems you have – there are so many of them already – but if you do not trust yourself and your own assessment, you will not proceed with the patient and a lot of unnecessary things will be done. (IP10)

#### Lectures

Education in the form of lectures—at the regional level, targeting all physicians—was perceived by some as influencing LVC use. The perceived influence of these lectures differed among the managers. Regional lectures were perceived as influencing the use of LVC because they were often based on clinical decision support, which describes both what should be done and what should not be done.

The theme was use imaging correctly and appropriately. It was held by a specialist in radiology and covered both when you should order an x-ray and when you should not. (IP8)

In contrast, they were also perceived as having no LVC content:

It has not been an explicit theme. We focus on diagnoses; we rarely focus on LVC. (IP3)

#### Quality assurance systems

Several quality assurance systems were described in the interviews as strategies that were or could be used to reduce LVC. All centers had access to the systems and could review their data as often as they liked to improve their performance. Examples of data included how many patients they had with different diagnoses, how many prescriptions of certain medications were ordered, and how many of a specific lab test had been ordered. They also had access to benchmark data on the different results, making it possible to compare their results with other centers both in the county and nationwide. Most performance measurements were related to a general goal of improving care. Managers differed in how often they reviewed their own data and how they used it. One common method was to review all data once per year, choose one measurement that they wanted to improve, and include it in the scorecard. Based on the chosen results, the center staff could also make a performance improvement plan. However, the work related to the quality assurance systems rarely focused on LVC. Still, the quality assurance systems involved many different variables so it would be possible to use the system to support reduced use of LVC.

You can pick an example and compare your data to a benchmark for all health care centers. I believe that it is a very successful strategy for a discussion. Like if we look at the lab test sedimentation rate, and then find that wow, we really order so many more of those tests than what is normal for all centers. (IP7)

#### Financial system

The financial system also had the potential to influence LVC use. Many of the managers described that the previous system, under which the centers were mainly paid for patients’ visits to a physician, had steered the centers toward always booking the patients with physicians when they contacted the center. This was described as increasing the use of LVC, since the physicians’ main tools for managing patients’ symptoms are lab tests, examinations, and medications.

When they reformed the system, we got paid so much more for visits to the physician than a visit to the nurse. This worked as an incentive to make all patients visit the physician, even for things that were not so important. When the patient met the physician, a throat test was ordered even though it was not needed, or maybe antibiotics were prescribed even though the patient did not need them. (IP1)

When the financial system changed to mainly reimburse the centers for the number of listed patients, the incentive to schedule all patients with a physician decreased. However, this system had its disadvantages. Reimbursing the centers for the number of listed patients led to an increased emphasis on having satisfied patients who remained listed at the center. Concerns were expressed that this created an incentive for physicians to act in accordance with patient requests for specific examinations or medications to reduce the risk of losing them as listed patients.

When patients are expected to list themselves at the center, it implies that we should be available and accommodating, and there is a slight risk that this will lead to a drift (in following guidelines), especially when it concerns something that is harmless for the patient: to order that lab test, conduct that examination or prescribe that medication. (IP6)

Another LVC-relevant aspect of the financial system was the monitoring of examination and lab test costs. This monitoring encouraged the centers to try to reduce them by discussing them on a regular basis. This surveillance could potentially help reduce the use of lab tests in general, including LVC lab tests.

…and of course, lab tests, since it is an expensive cost for the center, this is something that is being looked at. (IP2)

#### Center-specific strategies

Three strategies were found on a center level: process strategies, locally held lectures, and discussions about guidelines. Process strategies included different ways of changing work processes to influence the way each center functioned. These strategies were developed at each center by the manager in collaboration with the personnel. The examples described by the informants were often directly aimed at reducing LVC. Some of the process strategies were influenced by regional strategies (i.e., scorecards, decision support, and financial system), but some were designed based on the initiatives of the managers or center personnel. One example of a process strategy was to stop scheduling patients with a physician when other professional categories were better options. It was argued that scheduling them to see another profession such as a nurse or a physiotherapist reduced the likelihood of the patient receiving LVC, as the other professions did not have access to ordering tests and examinations or prescribing medications.

We try to work a lot with who the patient should meet first and try to avoid it being the physician, since they are so good at ordering lab tests and prescribing medications or sick leave, and many things that are not in the patients’ best interest. (IP6)

They also had different processes for changing routines to reduce the use of specific LVC practices. Examples of routines included restricting what examinations junior physicians could order without approval from a senior physician, and changes in standard test ordering forms that created hurdles for the physicians to order examinations and tests that were considered of low value.

The normal lab tests that we use at the center are grouped in the ordering system to make it more efficient. For this to work, we need to have a couple of people who review these grouped tests and remove those that are no longer needed. (IP2)

A more general strategy to reduce or avoid LVC was to work toward continuity in contact between the physician and the patients by making sure that the center had a good work environment. This would reduce staff turnover and make it easy to recruit highly skilled professionals.

I can tell you right away that the most important thing is that you have a physician who is a specialist within primary care, who knows his or her patients and who is experienced in examining patients and can trust their own findings. That is what it is all about. That means education, experience, and continuity in relation to patient contacts. (IP10)

Locally held lectures included sharing of knowledge related to LVC that individuals had acquired and invited experts to present at their meeting. These lectures were perceived to influence the use of LVC. Some of the lectures were influenced by the regional strategies (lectures and guidelines) whereas others were planned entirely based on local initiatives.

Discussions about guidelines were the final local strategy that was center specific. Several of the centers described holding regular meetings within the entire personnel group or among the physician group to discuss how to manage different diagnoses. Those discussions included what they should do and what they should not do (i.e., LVC issues).

We also have a lot of discussions within our center on how we manage (different patient symptoms), what we should manage, and we use each other for help. (IP9)

Some of the discussions were based on the personnel’s own clinical experience whereas others were in relation to guidelines.

When we have meetings, we discuss how to manage different patient cases and look at the guidelines.

### Deductive analysis of the mechanisms involved in the strategies

To understand the mechanisms involved in the strategies, we analyzed the above LVC-related strategies in terms of the three-term contingency and rule governing.

#### Three-term contingency

None of the five strategies decided on a regional level could be interpreted as influencing antecedents or consequences in the physicians’ environments directly. The only way for these strategies to change the direct contingencies would be via changes at the centers. For instance, feedback from the scorecards on costs related to lab tests did not directly impact the physicians’ ordering of these tests but encouraged centers to develop their own strategies to reduce costs. These could then impact physicians in turn. The same type of process applied to all management strategies developed at a regional level, including guidelines and education.

Contrary to the regionally developed ones, the strategies developed by the centers could be interpreted as influencing direct contingencies for the physicians. For instance, removing LVC lab tests from standard ordering sets and not permitting junior physicians to sign their own imaging orders made it difficult to use these practices. These changes were similar to the behavior analysis principle of the increased response effort, which increases the amount of effort needed to perform a behavior related to a reinforcing consequence. Scheduling patients with professions other than physicians removed the situation (i.e., the patient interaction) and thus the contingencies. Planning for continuity in contact between the physician and the patients could reduce the probability of adverse reactions from the patients when saying no to prescribing LVC. All strategies developed by the centers could be interpreted as aiming to reduce behaviors related to LVC use rather than increasing behaviors related to not using LVC.

#### Rule-governing behavior

Three strategies could be interpreted as trying to influence behavior through rule governing. The regionally developed strategies, lectures, and guidelines, as well as center-specific lectures and discussions on how to reduce the use of specific LVC practices, all included instructions on what not to do with an implied consequence to improve patient health. However, this consequence was not always sufficiently immediate for a physician to experience (i.e., the effects of not using LVC are not always noticeable from a short-term perspective). The lack of immediate consequences for following the rule, such as experienced health improvement for the patients, decreases the probability of guidelines and education acting as rules governing the behavior. The locally held lectures and discussions about guidelines provided socially mediated consequences for following the rules and can thus be interpreted as having a stronger potential for functioning as a rule.

**Fig. 1:**
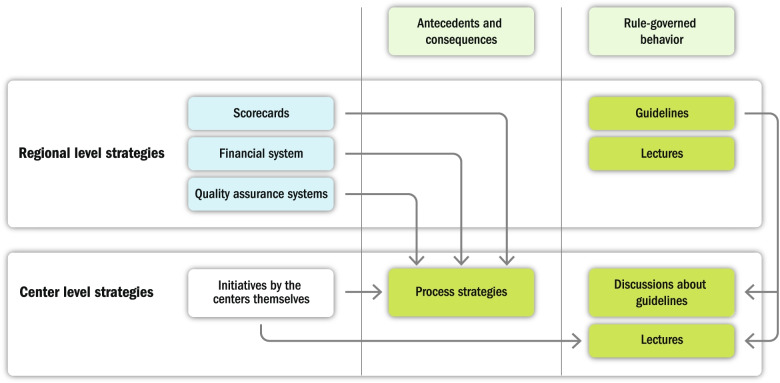
Strategies divided based on where they were initiated (regional or center level) and by their function on influencing behavior (antecedents and consequences or rule-governed behaviors)

All strategies are illustrated in Fig. 1 and are divided both based on which level of the organization each strategy was initiated (regional or center level) and in what way they influenced LVC-related behaviors (antecedents and consequences or rule-governed behavior).

## Discussion

We identified a total of eight management strategies that dealt with LVC de-implementation, whether directly or indirectly. Five of those were developed at the regional level with a more general goal of improving care, and thus not with de-implementation of LVC as their main purpose. Three types of strategies: process changes, locally held lectures, and discussions about guidelines, were developed at the center level and often with the main purpose of de-implementing LVC. Regarding the mechanism of these management strategies, we found that all strategies were targeted at decreasing behaviors related to the provision of LVC; none aimed to increase behaviors related to not using LVC. Furthermore, our applied behavior analysis revealed that the regionally developed strategies did not influence the physicians’ behavior by changing antecedents or consequences in their environment directly, but through a chain of behaviors where scorecards, financial follow-up, and quality improvement systems acted as antecedents for the centers to develop local processes to influence their results. This finding implies that one way of influencing de-implementation on a system level could be by encouraging centers to develop local strategies. We also found three strategies that could impact behaviors through the mechanism of rule governance. The center-specific strategies were partly developed as a result of regional strategies and partly as a result of local initiatives. Center-specific strategies had a particularly strong potential for impacting LVC use and functioned both through changing antecedents and consequences and by fostering rule-governed behavior. We discuss these findings and their potential contribution to implementation science and practice below.

The five regional strategies we identified—scorecards, guidelines, education, financial follow-up, and quality improvement systems—have been mentioned in prior de-implementation research [30]. Despite their more general purpose (i.e., as opposed to a specific de-implementation purpose), they were perceived as influencing behaviors related to the use of LVC. However, different centers appeared to focus on different LVC practices and used different strategies to reduce them, seemingly based on arbitrary reasons. Thus, decisions regarding which LVC practice to reduce and what strategies should be used to achieve this reduction were not based on thorough center-level analyses.

Center-specific strategies focused more directly on reducing LVC use by changing local patient care processes, host local lectures on LVC, or have discussions about guidelines. Redesigning processes has been suggested as an important component related to implementation [31] and could be considered relevant also in reducing LVC use as well.

Our deductive analysis based on applied behavior analysis revealed that only the center-specific process strategies involved changing the physicians’ direct environments, and thus changing antecedents and consequences related to LVC. This can be an effective approach, as contingencies may vary considerably between different environments and management strategies need to be designed by analyzing the existing circumstances which have been shown in previous studies [7]. The center-specific strategies mainly focused on making it more difficult to provide a given LVC (i.e., increasing the response effort for ordering a lab test or an examination) or removing the situation entirely by scheduling patients with other professions. Thus, we found no strategies focused on providing support or encouragement for behaviors related to not using an LVC (i.e., differential reinforcement [14]). Examples of behaviors related to not using LVC could be to make the decision to not use a practice, to communicate to a patient the lack of benefit of a practice, or to recommend self-care strategies to patients who do not need any medical treatment. The lack of strategies aiming at supporting not using LVC could imply that such strategies are considered to be difficult to develop. From the perspective of applied behavior analysis, perspective lack of these types of strategies represents a lost opportunity for supporting physicians in de-implementing LVC. Our findings highlight the importance of research in implementation science for understanding how management strategies focusing on increasing behaviors related to not using an LVC can be developed.

We also found strategies aiming to impact physicians’ behaviors through rule governance both at regional (i.e., guidelines and lectures) and center (i.e., locally held lectures and discussion forums on guidelines) levels. According to the principles of rule-governed behavior, the strategies developed on a regional level could have been improved to give a clear statement (instead of an implied one) of the expected consequences for not using LVC. The center-level strategies that functioned as governing rules were the only ones that seemed to provide socially mediated consequences for following the rules. This means that the forums showed especially good potential for influencing behaviors related to de-implementation.

Our use of applied behavior analysis as a method for analyzing the results contributed to an understanding of mechanisms involved in management strategies to de-implement LVC. Applying this approach provides an important step in explaining whether and how different management strategies can influence behaviors related to the use of LVC. Using applied behavior analysis allowed a theoretical understanding of how management strategies might potentially work before they are used in practice. Furthermore, this analysis can be used to understand why certain strategies did or did not have desired results after testing.

### Strengths and limitations

There are some limitations to our study that need to be considered when interpreting the findings. The study was conducted during the COVID-19 pandemic, which made it difficult to recruit as many participants as originally planned. Still, research suggests that the more information power, i.e., information relevant for a study, a sample holds, the fewer interviews are needed. Information power depends on the study aim, sample specificity, use of an established theory or framework, quality of the dialogue of the interviews, and analysis strategy [27]. According to Guest et al. [32], 12 interviewees should be sufficient if the informants are knowledgeable about the subject, data quality is satisfactory, and the aim is to understand common perceptions and experiences rather than to assess the variation between the groups. It is noteworthy that the two final interviews did not provide any contradictory or new data; this can be interpreted as the data being trustworthy. The situation for the health care centers was also influenced by the pandemic in terms of transitioning to more digital patient visits and other types of prioritizing. During the data collection period, most centers were preoccupied with coordinating vaccinations for their patients who were at risk. This may have influenced their opportunity to focus on working with quality improvement in general and reducing LVC in particular. Drawing conclusions on the mechanisms involved in management strategies based on interviews should always be done with caution. Individual practitioners are the only ones who behave in a situation where LVC is used. They are the ones being influenced by different types of contingencies, and exactly which contingency can explain a given behavior is difficult to determine based on interviews with their managers. One can always wonder whether their descriptions of influence are those that accurately explain the behaviors.

The study also has strengths. Our multidisciplinary research team enhanced the credibility of the study: the composition of the team allowed different perspectives on the topic of LVC de-implementation. To our knowledge, behavior analysis has never been used empirically to understand management strategies to de-implement LVC. Behavior analysis is useful when trying to track a process of influence from overarching strategies to specific influence on the individuals deciding whether to use an LVC practice.

## Conclusions

We identified eight different management strategies intended to influence LVC. Five of the strategies developed at the regional level had the potential to influence physicians’ LVC-related behaviors, either by functioning as rules with which to de-implement LVC or by initiating local strategies at each center that in turn influenced LVC use. The local strategies had greater potential for influencing de-implementation, even though some of them were likely initiated as an effect of strategies on a regional level. Both strategies on a system level (council level) and specific strategies on a local level (health care center level) must be taken into consideration to influence LVC-related behaviors. Center-level strategies have a specific opportunity to impact LVC-related behaviors because they can be tailored to specific circumstances. Using applied behavior analysis to understand these circumstances can help tailor strategies to reduce the use of LVC.

## Supplementary Information

**Additional file 1:** **Table S1.** COREQ (COnsolidated criteria for REporting Qualitative research) Checklist.

**Additional file 2.** Interview guide step 1.

**Additional file 3.** Interview guide step 2.

## Data Availability

The datasets used will be available from the corresponding author on reasonable request.

## Abbreviation

LVC: Low-value care
