# Supplementary material for: Management strategies to de-implement low-value care—an applied behavior analysis
Source: Implement Sci Commun. 2022 Jun 25;3:69. doi: 10.1186/s43058-022-00320-3 (PMC9233807; doi:10.1186/s43058-022-00320-3)
Supplement: Supplementary file 2 — Additional file 2. Interview guide step 1. [file 43058_2022_320_MOESM2_ESM.docx]

Interview guide step 1

**The first qustion is a more general question concerning de-implementation of LVC within health care. What are your thoughts on the topic (main question in bold letters, possible follow up questions in normal letters) ?**

Positive?
Negative?

**Is this something that you think about during your everyday work?**

What can initiate these thoughts?

If, then how do you handle it? What do you do in practice?

Can you give any specific examples? How did it work? What did you do?

**Do you have any management strategies with a purpose of of influencing how the different centers works with this issue?**

Formal management strategies (performance indicators, monitoring, incentives)

Strategies to influence knowledge (education, quality indicators)

What’s the origin of these strategies? (Planned them yourself/other planned them)

**Are any of these strategies related specifically to influence the work of physicians?**

**How do you perceive that the management strategies work?**

Can you give a specific example?

**Who do you believe should be responsible for this kind of issues?**

Regional, center or individual level?

**What other management strategies do you use (not related to LVC)?**

**Are any of these related specifically to the work of physicians?**

Formal management strategies (performance indicators, monitoring, incentives)

Strategies to influence knowledge (education, quality indicators)

What’s the origin of these strategies? (Planned them yourself/other planned them)

**How do you perceive that the more general management strategies work?**

**Have you noticed examples were the general managment strategies influences the use of LVC?**

**If they do, in what way?**

Something that you would like to add that I haven’t asked about?
