# Supplementary material for: Management strategies to de-implement low-value care—an applied behavior analysis
Source: Implement Sci Commun. 2022 Jun 25;3:69. doi: 10.1186/s43058-022-00320-3 (PMC9233807; doi:10.1186/s43058-022-00320-3)
Supplement: Supplementary file 3 — Additional file 3. Interview guide step 2. [file 43058_2022_320_MOESM3_ESM.docx]

Interview guide step 2

**The first qustion is a more general question concerning de-implementation of LVC within health care. What are your thoughts on the topic (main question in bold letters, possible follow up questions in normal letters) ?**

Positive?
Negative?

**Is this something that you think about during your everyday work?**

What can initiate these thoughts?

If, then how do you handle it? What do you do in practice?

Can you give any specific examples? How did it work? What did you do?

**I am now going to present a couple of management strategies that could influence the use of LVC.**

1. Scorecards

Are you familiar whith them?
Do you receive any feedback based on them?
How do you use them at the center?
How do they work in practice (Easy/difficult to use/influences LVC)?

1. Decision support

Are you familiar whith them?
Do you receive any feedback based on them?
How do you use them at the center?
How do they work in practice (Easy/difficult to use/influences LVC)?)

1. Education

Are you familiar whith them?

Do you receive any feedback based on them?

How do you use them at the center?

How do they work in practice (Easy/difficult to use/influences LVC)?

1. Quality assurance systems?

Are you familiar whith them?

Do you receive any feedback based on them?

How do you use them at the center?

How do they work in practice (Easy/difficult to use/influences LVC)?

**Who do you believe should be responsible for this kind of issues?**

Regional, center or individual level?

**What other management strategies do you use (not related to LVC)?**

**How do you perceive that the more general management strategies work?**

**Have you noticed examples were the general managment strategies influences the use of LVC?**

**If they do, in what way?**

Something that you would like to add that I haven’t asked about?
